# High-efficiency Fresnel zone plates for hard X-rays by 100 keV e-beam lithography and electroplating

**DOI:** 10.1107/S0909049511002366

**Published:** 2011-03-10

**Authors:** Sergey Gorelick, Joan Vila-Comamala, Vitaliy A. Guzenko, Ray Barrett, Murielle Salomé, Christian David

**Affiliations:** a Paul Scherrer Institut, CH-5232 Villigen, Switzerland; b European Synchrotron Radiation Facility, BP 220, F-38043 Grenoble Cedex, France

**Keywords:** Fresnel zone plate, hard X-rays, X-ray optics, electron-beam lithography, Au electroplating, PMMA

## Abstract

The fabrication and characterization of Fresnel zone plates (FZPs) for hard X-ray microscopy applications are reported. High-quality 500 nm- and 1 µm-thick Au FZPs with outermost zone widths down to 50 nm and 70 nm, respectively, and with diameters up to 600 µm were fabricated. The diffraction efficiencies of the fabricated FZPs were measured for a wide range of X-ray energies (2.8–13.2 keV) showing excellent values up to 65–75% of the theoretical values, reflecting the good quality of the FZPs. Spatially resolved diffraction efficiency measurements indicate the uniformity of the FZPs and a defect-free structure.

## Abstract 2

The efficiencies of several Fresnel zone plates, that were fabricated using a direct-write method with high-energy electrons, were measured over a wide range of photon energies.

## 1. Introduction

X-ray microscopy has the potential for imaging thick samples with high resolution owing to the high penetration power of X-rays and their short wavelength.^1^ To fully exploit the capabilities of X-ray microscopy, it is necessary to efficiently control X-ray beams. Among a variety of techniques to manipulate the X-rays (*e.g.* for focusing or sample illumination), diffractive optical elements are some of the most versatile. For instance, Fresnel zone plates (FZPs) can routinely focus soft X-rays into spots smaller than 50 nm and even resolve sub-10 nm features (Vila-Comamala *et al.*, 2009 ▶). The resolution performance of FZPs is proportional to the width of the outermost ring or ‘zone’. To produce FZPs for high-resolution applications, high-resolution lithography methods are generally used. The method of choice is typically electron-beam lithography (EBL) which is, in principle, capable of sub-10 nm patterning. However, patterning FZPs by EBL with the smallest outermost zone width is not sufficient because the efficiency of FZPs at a given photon energy, that is the ratio of the focused to the incident intensity, depends on the height of the zones, as well as the material from which they are made. Ideally, the zones must be sufficiently tall to provide a phase shift close to π for the highest diffraction efficiency (DE). For soft X-rays the required heights are typically of the order of a few hundreds of nanometers, while for the hard X-rays the optimal zone height would be several micrometers even for high-*Z* materials with high refractive index (*e.g.* Au). Thus, to provide high-efficiency focusing of hard X-rays into a small spot, FZPs must have nanostructured zones with extremely high aspect ratios. The conventional low-energy EBL is capable of patterning only shallow nanostructures owing to the strong scattering of electrons and must, therefore, be followed by post-processing steps, *e.g.* several etchings and electroplating (Schneider *et al.*, 1995 ▶; Jefimovs *et al.*, 2007 ▶; Feng *et al.*, 2007 ▶; Lindblom *et al.*, 2007 ▶), to transfer the low-aspect-ratio structures produced by EBL in thin layers of photoresist into taller metallic nanostructures. The fabrication of FZPs with high-aspect-ratio zones is, therefore, challenging. A much faster and more reliable approach is to use 100 keV EBL to directly write high-aspect-ratio nano­structures in thick layers of resist, which can be used as a mold for electroplating to produce metallic FZPs (Lo *et al.*, 2007 ▶; Chen *et al.*, 2008*a* ▶,*b* ▶; Gorelick *et al.*, 2010*a* ▶,*b* ▶). Unlike low-energy electron beams (<30 keV) of conventional e-beam writers, high-energy, 100 keV, electrons are able to penetrate several micrometers into the resist with little scattering, making them suitable for exposure of high-aspect-ratio nanostructures in polymethyl-methacrylate (PMMA) resist. Such a direct-write approach eliminates the need of optimization of several consecutive nanofabrication steps, thus increasing the reliability of the fabrication process and its yield. Using this direct-write approach we fabricated FZPs made from Au by filling the PMMA molds with Au by electroplating.

In addition to the height of the zone, the pattern accuracy is a prerequisite of the high-efficiency focusing by a FZP. Thus, if the line-to-space ratio of a FZP is not optimal (*e.g.* 0.5 for the outermost zones), the photons are rechanneled from the first diffraction order^2^ to other orders, thus decreasing the first-order focus efficiency (Kirz, 1974 ▶). We optimized the fabrication parameters (exposure, development time and line shrinkage with respect to the target line width) such that the almost optimal pattern dimensions were realised in fabricated FZPs.

The efficiencies of several fabricated FZPs were measured over a wide range of energies (2.8–13.2 keV). To the best of our knowledge this is the first measurement of the efficiencies of various FZPs in such a wide range of photon energies. The measured DEs are relatively close to the theoretical values, reflecting the good quality of the FZPs. Such measurements are important for assessment of the quality of the FZPs and further optimization of the fabrication parameters.

## 2. Fabrication process

**Figure 1:**
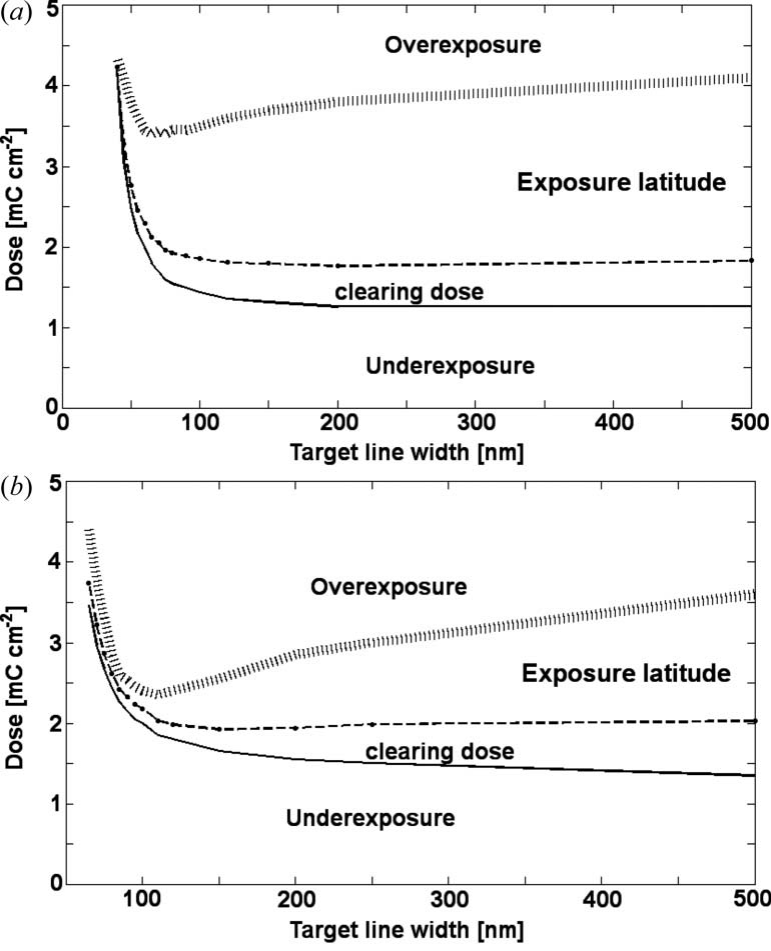
Area doses and exposure windows for dense lines exposed in PMMA for 30 nm and 40 nm shrinkages in (*a*) 540 nm-thick PMMA and (*b*) 1.1 µm PMMA. The dashed lines show the doses (typically 10–20% above the clearing doses) that were used in exposures of FZPs.

**Figure 2:**
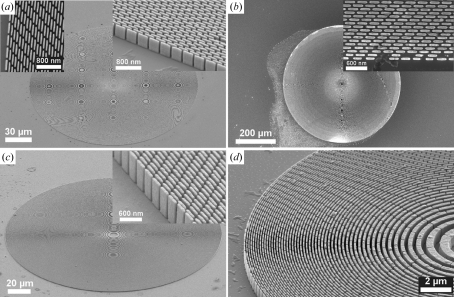
Scanning electron microscope images of Au FZPs fabricated by exposing (*a*, *b*) 540 nm- and (*c*, *d*) 1.1 µm-thick PMMA and filling the developed trenches with Au by electroplating. (*a*) 200 µm-diameter FZP with a 50 nm outermost zone. The inserts present the outermost regions (tilts 0° and 45°). (*b*) 600 µm-diameter FZP with a 50 nm outermost zone. The insert presents the outermost region. (*c*) 200 µm-diameter FZP with a 70 nm outermost zone. The insert presents the outermost regions (tilt 60°). (*d*) 30 µm-diameter FZP with a 70 nm outermost zone (tilt 55°).

The fabrication process started by vapor-coating Si_3_N_4_ membranes with a Cr/Au/Cr plating base (5/20/5 nm). The upper Cr layer was necessary to promote the adhesion of PMMA nanostructures, which otherwise adhered weakly to the Au surface. Resist layers were spin-coated on the membranes to yield 540 nm- or 1.1 µm-thick layers. The exposure of patterns was performed on a 100 keV electron-beam writer (Vistec EBPG 5000Plus). Exposed line dimensions were reduced by 30–40 nm with respect to the target line width and exposed at higher doses, such that after the development the target width was achieved. The exposure doses were empirically determined as a function of the line width such that the simultaneous development of all the zones across the FZP was ensured (Fig. 1 ▶). The chips were developed in an IPA:water (7:3) mixture for 10 s and 20 s for 540 nm- and 1.1 µm-thick PMMA layers, respectively. After dry etching the upper Cr layer the patterns were transferred into Au by electroplating at 2.5 mA cm^−2^ (direct current) in a cyanide-based plating bath. More details on the optimization of the dose-shrinkage-development parameters can be found elsewhere (Gorelick *et al.*, 2010*a* ▶). Fig. 2 ▶ shows some of the fabricated FZPs. The structures are uniform and the overall quality of the devices is exceptionally good. Both large diameters [up to 600 µm; Fig. 2(*b*) ▶] and small diameters down to 30 µm [Fig. 2(*d*) ▶] and 20 µm (Gorelick *et al.*, 2010*a* ▶) were realised. During the exposure of the FZPs, we typically varied the doses across the FZP patterns as a function of the line width such that the doses remained within the exposure latitudes (Fig. 1 ▶). We tried to keep the exposure doses as close as possible to the clearing doses, that is to the interface between underexposure and the exposure latitude (Fig. 1 ▶); however, we noticed that often the patterns in PMMA were not fully developed, presumably owing to uncertainties in the developer concentration and its temperature. By using higher doses, typically 10–20% above the clearing doses (Fig. 1 ▶), we avoided this problem leading to fabrication yields close to 100%. In addition, rather than exposing continuous lines in PMMA, we exposed interrupted lines which were later translated into interrupted Au zones in FZPs (Fig. 2 ▶). This segmentation was necessary to improve the mechanical stability of high-aspect-ratio PMMA mold which tended to collapse during the drying process owing to the action of capillary forces of the liquid trapped between continuous lines. To assess the quality of the fabricated zone plates we measured their diffraction efficiencies. This information is useful for understanding the effects of processes parameters, such as exposure doses and the line segmentation, on the performance of the FZPs in order to further optimize the fabrication process, and consequently fabricate the most efficient X-ray diffractive lenses.

## 3. DE measurements

**Figure 3:**
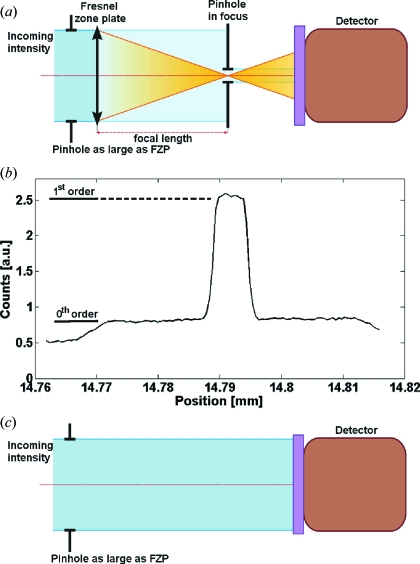
DE measurement of FZPs having different outermost zone widths *dr*, heights *H* and diameters *D*. (*a*) The pinhole is scanned through the focus of a zone plate illuminated through an aperture having the same diameter as the zone plate. (*b*) Resulting profile of the measurement. (*c*) A reference measurement of the beam intensity is taken by removing the pinhole and the zone plate from the beam.

**Figure 4:**
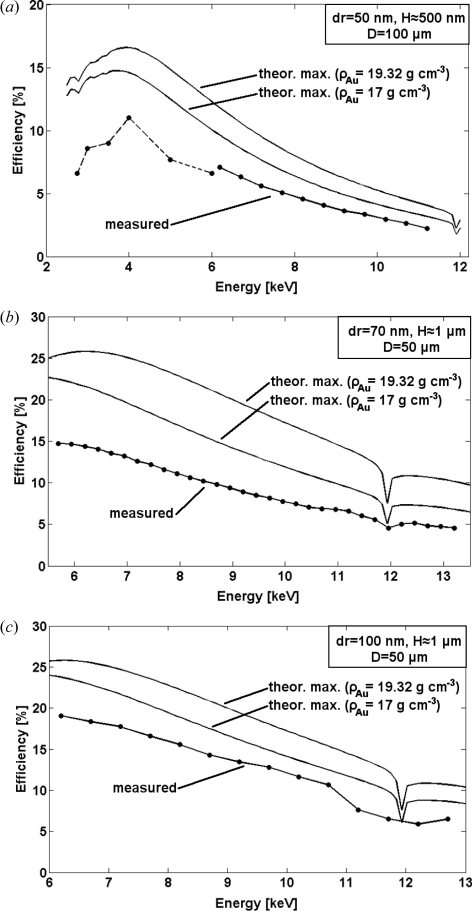
Diffraction efficiencies (first order) of various FZPs with an outermost zone width *dr*, diameter *D* and zone height *H* measured over a wide range of X-ray energies and compared with the theoretical maximum values calculated from the tabulated X-ray optical constants. The interruption in the measured efficiencies in (*a*) represents a change of the experimental set-up for measurements at photon energies below 6 keV (see text).

The DE measurements for energies above 6 keV were performed at the ID06 beamline of the European Synchrotron Radiation Facility (ESRF). The measurements for energies below 6 keV were performed at the ID21 beamline of the ESRF. A schematical presentation of the measurement process is shown in Fig. 3 ▶. The DE measurement was performed by scanning a 5–10 µm order-selecting aperture (OSA) through the first-order focal spot of a FZP that was illuminated through an aperture having the same diameter as the zone plate (Fig. 3*a* ▶). Both vertical and horizontal scans across the focus were performed for control and verification of the scan results. A typical result of such a scan is presented in Fig. 3(*b*) ▶. The focused intensity was calculated by subtracting the background signal caused by the zeroth-order radiation. Next, both the FZP and OSA were removed from the beam and the intensity of the incident beam was measured through the upstream aperture (Fig. 3*c* ▶). The DE was estimated as the ratio of the focused to incident intensity. The measured efficiencies for three different types of zone plates are presented in Fig. 4 ▶. Generally, for 500 nm-thick FZPs the efficiencies are ∼60% of the theoretical maximum values calculated from the tabulated X-ray optical constants (Henke *et al.*, 1993 ▶), approaching ∼75% for the 1 µm-thick FZPs with a 100 nm outermost zone. The discrepancy between the theoretical and measured values cannot solely be explained by the presence of the interruption of the zones. The phase-shifting area ‘lost’ to the interruptions accounts only to 3%, 5.5% and 7.2% of the measured FZPs with 100 nm, 70 nm and 50 nm outermost zones, respectively. One of the main factors contributing to the reduction of the DE is the lower density^3^ of electroplated Au of 17 g cm^−3^ compared with 19.3 g cm^−3^ for bulk Au. Fig. 4 ▶ compares the theoretical maximum DE values for FZPs made from bulk and electroplated Au with a reduced density. This density decrease results in a reduced phase-shift of the incoming X-ray beam and consequently to the reduced DE. The corresponding decrease in the DE is ∼15% for the measured energy range (Henke *et al.*, 1993 ▶). The zone segmentation and the reduced density of the electroplated Au thus together result in an 18–22% loss of the DE, while the remaining 15–20% loss can be attributed to the uncertainty in the height of the electroplated structures and the zone width, which can vary to up to 10% from the optimal line width (Gorelick *et al.*, 2010*a* ▶). Nevertheless, the effect of line segmentation on the DE of a FZP is manifested in a systematically reduced overall DE with increased density of segmentation. Another factor that can potentially contribute to the DE loss is the diffuse scattering of X-rays from the interruptions between the zone segments. The effect of this scattering is, however, difficult to assess owing to the complexity of the FZP/buttressing geometry and hence the required substantial computational effort.

FZPs that were exposed to increased doses had typically broadened zones, and hence non-optimal duty cycle of the gratings, and their measured efficiencies were systematically lower than those of the FZPs that were exposed at lower doses (not shown). We, therefore, conclude that using the lower exposure doses in our FZPs fabrication process is optimal. Further research and development of electroplating bath chemistry and plating conditions are required to optimize the density of the deposited Au which can lead to the fabrication of FZPs with efficiencies approaching the theoretical maximum values.

## 4. Spatially resolved DE

**Figure 5:**
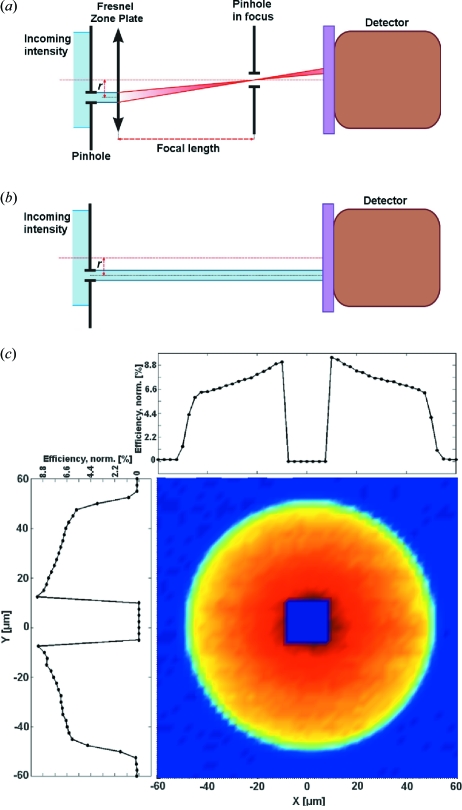
Spatially resolved DE measurement. (*a*) The FZP is illuminated through a pinhole and the focused intensity is filtered by an OSA in the focus of the FZP. (*b*) The FZP and OSA are removed and the incoming intensity is measured. (*c*) Spatially resolved diffraction efficiency of a 500 nm-thick 100 µm-diameter FZP with an outermost zone of 50 nm at 6.2 keV photon energy.

While the DE measurements aid in assessing the overall performance of a FZP, spatially resolved DE measurements help to estimate the contributions of different parts of the FZP to the total DE and reveal defects, *i.e.* voids in the electroplated structures. Spatially resolved DE measurement of a 500 nm-thick and 100 µm-diameter FZP with an outermost zone of 50 nm was performed at 6.2 keV photon energy. The measurement was performed by illuminating the FZP through a 5 µm pinhole and selecting the focused intensity through a 5 µm OSA positioned in the focus of the FZP [Figs. 5(*a*) and 5(*b*) ▶]. By scanning the upstream pinhole a map of local DE was collected. The result of such a measurement is presented in Fig. 5(*c*) ▶. The measurements for the central area of the FZP were numerically removed from the plot, as the diffracted radiation signal was dominated by the strong zeroth-order photons when the axis of the pinhole and the OSA coincided with the centre of the FZP (Fig. 5*a* ▶). The profile of the local diffraction intensities is remarkably uniform which indicates a defect-free FZP. The local diffraction intensity decreases toward the edges of the FZP (Fig. 5*c* ▶). This is consistent with the previously reported measurements of the local DE that was shown to depend on the variation in the plated height of the zones, line-to-space ratio and roughness, and to typically have a maximum at the center of a FZP dropping toward the edges (Bertilson *et al.*, 2007 ▶). Scanning electron microscope inspection of the measured FZP, however, did not reveal significant variations of the zone height and line-to-space ratio. The obtained profile of the local DE is consistent with the design of the FZP which has radially increasing density of the zone interruptions that were necessary to support the thinner outermost PMMA zones. In the studied FZP the phase-shifting area ‘lost’ owing to the interruptions in the outermost zone accounts to 21% of the zone area. While for the inner zones, where a few interruptions are found, the local DE is 0.088, the local DE of the outermost zones is 25% lower at 0.066. This is in good agreement with the loss of DE associated with the zone interruptions, further indicating the good quality and height uniformity of the electroplated FZPs. Decreasing the buttressing density, especially of the outer zones, would improve the local DE and consequently the total efficiency of the FZP.

## 5. Conclusion

We have developed a fast and reliable fabrication method of high-quality FZPs for multi-keV X-rays. The diffraction efficiencies of several FZPs were measured over a wide range of X-ray energies to assess the quality of the FZPs and the influence of various fabrication parameters on the DE. The measured diffraction efficiencies are 60–80% of the theoretical values. The main cause of the DE loss as compared with the theoretical maxima is the density of electroplated Au. The effect of buttressing of polymer mold was manifested in decreased local DE of the areas in the FZP having higher density of buttressing. The overall local DE is uniform which indicates a good quality of the FZP, height uniformity of the zones and lack of defects.

## Footnotes

1 For a recent overview of X-ray microscopy, we refer to the proceedings of the *9th International Conference on X-ray Microscopy* [*J. Phys. Conf. Ser.* (2009), **186**] and the proceedings of the *10th International Conference on X-ray Microscopy* (in the press).

2 Since the DE decreases proportionally to *m* ^−2^, where *m* is the diffraction order, typically the first diffraction order is used as it is the most efficient. The line-to-space ratio in a FZP can be optimized such that other diffraction orders, *e.g.* second or third, are enhanced; however, the requirements for the pattern accuracy are more stringent.

3 AUTRONEX-CC electrolyte data sheet, Enthone Corp.
